# Ventilatory support and hospital stay after liver transplant in cirrhotic patients with hepatopulmonary syndrome

**DOI:** 10.1590/S1679-45082017AO4081

**Published:** 2017

**Authors:** José Leonardo Faustini Pereira, Lucas Homercher Galant, Eduardo Garcia, Luis Henrique Telles da Rosa, Ajácio Bandeira de Mello Brandão, Cláudio Augusto Marroni

**Affiliations:** 1 Universidade Federal de Ciências da Saúde de Porto Alegre, Porto Alegre, RS, Brazil.

**Keywords:** Liver cirrhosis/etiology, Hepatopulmonary syndrome/complications, Respiration artificial, Length of stay, Noninvasive ventilation, Intensive care units, Cirrose hepática/etiologia, Síndrome hepatopulmonar/complicações, Respiração artificial, Tempo de internação, Ventilação não invasiva, Unidades de terapia intensiva

## Abstract

**Objective:**

To compare mechanical ventilation time, need for non-invasive ventilation, length of intensive care unit stay, and hospital stay after liver transplant in cirrhotic patients with and with no diagnosis of hepatopulmonary syndrome.

**Methods:**

This was a prospective cohort study with a convenience sample of 178 patients (92 with hepatopulmonary syndrome) who were diagnosed as alcoholic or hepatitis C virus cirrhosis. The statistical analysis included Kolmogorov-Smirnov test and Students *t* test. Data were analyzed using SPSS version 16.0, and p values <0.05 were considered significant.

**Results:**

Out of 178 patients, 90 underwent transplant (48 with no hepatopulmonary syndrome). The Group diagnosed with Hepatopulmonary Syndrome had longer mechanical ventilation time (19.5±4.3 hours *versus* 12.5±3.3 hours; p=0.02), an increased need for non-invasive ventilation (12 *versus* 2; p=0.01), longer intensive care unit stay (6.7±2.1 days *versus* 4.6±1.5 days; p=0.02) and longer hospital stay (24.1±4.3 days *versus* 20.2±3.9 days; p=0.01).

**Conclusion:**

Cirrhotic patients Group diagnosed with Hepatopulmonary Syndrome had higher mechanical ventilation time, more need of non-invasive ventilation, as well as longer intensive care unit and hospital stay.

## RESUMO

**Objetivo:**

Comparar tempo de ventilação mecânica, necessidade de uso de ventilação não invasiva, tempo de permanência na unidade de terapia intensiva e tempo de hospitalização após transplante hepático em cirróticos com e sem diagnóstico de síndrome hepatopulmonar.

**Métodos:**

Estudo de coorte prospectiva com amostra de conveniência composta por 178 pacientes (92 com síndrome hepatopulmonar) com diagnóstico de cirrose por álcool ou pelo vírus da hepatite C. A análise estatística foi realizada por meio do teste Kolmogorov-Smirnov e do teste *t* de Student. Os dados foram analisados pelo programa SPSS versão 16.0, e valores de p<0,05 foram considerados significantes.

**Resultados:**

Dos 178 pacientes, 90 foram transplantados (48 sem síndrome hepatopulmonar). O Grupo com Síndrome Hepatopulmonar apresentou maior tempo de ventilação mecânica (19,5±4,3 horas *versus* 12,5±3,3 horas; p=0,02), maior necessidade de uso de ventilação não invasiva (12 *versus* 2; p=0,01), maior permanência na unidade de terapia intensiva (6,7±2,1 dias *versus* 4,6±1,5 dias; p=0,02) e maior tempo de hospitalização (24,1±4,3 dias *versus* 20,2±3,9 dias; p=0,01).

**Conclusão:**

O Grupo com Síndrome Hepatopulmonar apresentou maiores tempo de ventilação mecânica, necessidade de uso de ventilação não invasiva, permanência na unidade de terapia intensiva e tempo de hospitalização.

## INTRODUCTION

Liver cirrhosis is characterized by diffuse replacement of the normal liver structure by nodules of abnormal structure, surrounded by fibrosis, and it is present at the final stage of a series of hepatic pathological processes resulting from several causes.^(^ ^1^ ^)^ Among their complications are metabolic changes associated with patient malnutrition, leading to loss of a large quantity of muscle mass, displaying changes in functionality, and a condition of physical inactivity. The association of all these factors has a negative influence on Activities of Daily Living (ADL) and on quality of life of this population.^(^ ^2^ ^-^ ^5^ ^)^

The treatment of cirrhotic patients is complicated and complex and should be comprehensive, with little perspective of a long survival. Liver transplantation (LTx) undoubtedly allows greater survival of these patients as well as lower expenses with treatment.^(^ ^6^ ^-^ ^8^ ^)^ Nevertheless, anesthesia and pain, especially in incisions close to the diaphragm region, such as those of the LTx, which are associated with a poor muscular and functional condition, can induce hypoxemia, decrease lung volume, increase the use of accessory breathing muscles, and generate atelectasis, thus compromising the postoperative period.^(^ ^9^ ^,^ ^10^ ^)^

Due to the fact of the liver exerting an important function in various processes of the human body, during the waiting period for the procedure, many liver disease patients develop changes that affect other organs, such as brain, kidneys and lungs. The hepatopulmonary syndrome (HPS) is one of these abnormalities, defined by a triad that involves liver diseases and/or portal hypertension, intrapulmonary vascular dilations and abnormalities in arterial oxygenation (arterial partial pressure of oxygen − PaO_2_ <70mmHg or alveolar-arterial gradient for oxygen − P(A-a)O_2_ >20mmHg in room air).^(^ ^11^ ^,^ ^12^ ^)^ Its prevalence varies between 4 and 47%, according to the population studied and to the criteria used to define arterial deoxygenation in intrapulmonary vascular dilations.^(^ ^13^ ^-^ ^19^ ^)^

The complexity of the LTx, the muscular behavior of this population, and the aggravating role that the HPS can play, guide the objectives of this project.

## OBJECTIVE

To evaluate and compare time of mechanical ventilation, need for use of noninvasive ventilation, length of stay in the intensive care unit, and length of hospital stay after liver transplantation of cirrhotic patients with and without a diagnosis of hepatopulmonary syndrome.

## METHODS

A prospective cohort study of 90 cirrhotic patients (48 with no HPS), followed up at the Liver Transplant Outpatient Clinic of the *Complexo Hospitalar Santa Casa de Misericórdia de Porto Alegre*, in Porto Alegre, Rio Grande do Sul, who had their functional condition assessed by means of a treadmill stress test, 6 minute walk test (6MWT), and respiratory muscle strength, for a maximal period of 30 days before the LTx. After performing these procedures, the patients were followed up until hospital discharge or death.

The exclusion criteria adopted were a significant prior obstructive ventilatory *deficit*, defined by the Tiffeneau Index <0.70, with a forced expiratory volume in the first second (FEV_1_) <80% of that predicted; a significant prior restrictive ventilatory *deficit*, defined by forced vital capacity (FVC) <70% of that predicted, or other comorbidities not related to liver disease; significant orthopedic and cognitive changes, absolute contraindications for the performance of submaximal testing, and the presence of an intracardiac shunt.

The project was approved by the Research Ethics Committee of the hospital complex under official opinion no. 331.068, CAAE: 15389313.4.0000.5335.

### Diagnosis of hepatopulmonary syndrome

The diagnosis of the HPS was made by following pre-established criteria, verified by means of a myocardial contrast echocardiography (MCE).^(^ ^11^ ^)^ Gasometric parameters - especially the P(A-a)O_2_ - were evaluated together with MCE, since the isolated analysis of PaO_2_ can underestimate the real grade of hypoxemia. Therefore, P(A-a)O_2_ >20cmH_2_O was defined as necessary, due to its better accuracy in previous studies.^(^ ^11^ ^,^ ^12^ ^)^

### Procedures performed

Manovacuometry, 6MWT, and the treadmill stress test were also done as per the methodology used in our previous study.^(^ ^20^ ^)^ The first followed the rules of the American Thoracic Society (ATS); the second, the system and values of normalcy recommended by the Brazilian Society of Pneumology and Phthisiology (SBPT); and the third, according to the regulations established by the American College of Sports Medicine (ACSM).^(^ ^21^ ^-^ ^24^ ^)^

### Data analysis

To verify the normalcy of the sample, Kolmogorov-Smirnov’s test was used. For comparison of the variables among the groups, Student’s *t* test was used.

Data were analyzed with the Statistical Package of the Social Sciences (SPSS), version 16.0, and the level of significance adopted was 5%, considered significant when p<0.05.

## RESULTS

The clinical and anthropometric characteristics of the population, as well as the performance on the physical tests, are described on table 1. No statistically significant differences were found between the two groups as to age, height, weight, score on the Model for End Stage Liver Disease (MELD), classification as per the Child-Pugh Score, etiology of the cirrhosis and smoking, spirometric variables, and important clinical variables related to the performance of the surgical procedure, such as mean pulmonary artery pressure and coagulation factors. The Group with Hepatopulmonary Syndrome displayed a smaller distance covered in the 6MWT (361.8±50.9 *versus* 410.5±91.4; p=0.001), worse oxygen consumption peak (15.3±2.1 *versus* 17.3±2.6; p=0.001), lowest maximal inspiratory pressure (-55.1±9.8 *versus* -74.2±13.9; with p=0.01), lowest maximal expiratory pressure (60.1±12.25 *versus* 76.8±14.7; p=0.01), lower PaO_2_ (68.9±9.3 *versus* 88.1±10.1; p=0.01), and higher P(A-a)O_2_ (23.9±1.72 *versus* 13.4±1.65; p=0.01).

**Table 1:** Anthropometric, clinical, and physical testing characteristics of the sample

Variable	HPS (n=42)	NHPS (n=48)	p value
Age, (years)	60.4±7.10	58.7±8.20	0.9
Height, (cm)	167.7±6.4	169.5±7.1	0.8
Weight, (kg)	72.6±10.8	71.3±9.7	0.9
MELD	17.2±2.1	17.0±3.0	0.9
Smoking, (n)	6	9	0.8
Mean pulmonary artery pressure, (mmHg)	18.5±7.3	19.1±8.8	0.7
International Normalized Ratio	1.11±0.5	1.12±03	0.9
6MWT, (m)	361.8±50.9	410.5±91.4	0.001
VO_2_ peak, (mL/kg)	15.3±2.1	17.3±2.6	0.001
PImax, (cmH_2_O)	-55.1±9.8	-74.2±13.9	0.01
PEmax, (cmH_2_O)	60.1±12.25	76.8±14.7	0.01
Child Pugh Score B, (n)	17	19	0.8
Child Pugh Score C, (n)	25	29	0.7
Hepatitis C virus cirrhosis, (n)	28	30	0.8
Alcoholic cirrhosis, (n)	14	18	0.8
PaO_2_, (mmHg)	67.9±9.3	89.1±10.1	0.01
P(A-a)O_2_, (mmHg)	22.9±1.72	13.4±1.65	0.01
FVC, % of predicted	95±17.4	97±15.3	0.7
FEV_1_, % of predicted	88±13.3	90±14.7	0.7
FEV_1_/FVC	0.77±0.03	0.78±0.05	0.8

HPS: patients with hepatopulmonary syndrome. NHPS: patients with no hepatopulmonary syndrome; MELD: Model for End Stage Liver Disease; 6MWT: six-minute walk test; VO_2_: oxygen consumption; PImax: maximum inspiratory pressure; PEmax: maximum expiratory pressure; PaO_2_: partial arterial oxygen pressure; P(A-a)O_2_: alveolar-arterial gradient for oxygen; FVC: forced vital capacity; FEV_1_: forced expiratory volume in the first second.

As per the criteria of Porres-Aguilar et al.,^(^ ^12^ ^)^ of the 42 patients with HPS, 26 were classified as moderate (PaO_2_ ≥60mmHg and <80mmHg), 11 as light (PaO_2_ ≥80mmHg), and 5 as severe (PaO_2_ ≥50 and <60mmHg).

Table 2 shows variables in reference to the procedure, ventilatory support, and length of hospital stay. No statistically significant differences were found relative to ischemia time of the organ to be transplanted, operative time, and mortality during the period of 30 days, but patients with HPS remained for more hours under mechanical ventilation (19.5±4.3 hours *versus* 12.5±3.3 hours; p=0.02), had longer Intensive Care Unit stay (6.7±2.1 days *versus* 4.6±1.5 days; p=0.02), longer hospital stay. (24.1±4.3 days *versus* 20.2±3.9 days; p=0.01), and required more noninvasive ventilation (12 *versus* 2; p=0.01).

**Table 2:** Characteristics of liver transplant, ventilatory support, and hospitalization

Variable	HPS (n=42)	NHPS (n=48)	p value
Operative time, (hours)	4.2±1.2	4.5±1.0	0.9
Organ ischemia, (hours)	6.5±2.1	6.3±2.5	0.9
MV, (hours)	19.5±4.3	12.5 ±3.3	0.02
ICU, (days)	6.7±2.1	4.6±1.5	0.02
Hospital, (days)	24.1±4.3	20.2±3.9	0.01
Use of NIV, (n)	12	2	0.01
Death within 30 days, (n)	2	2	0.9

HPS: patients with hepatopulmonary syndrome; NHPS: patients with no hepatopulmonary syndrome; MV: mechanical ventilation; ICU: intensive care unit; NIV: noninvasive ventilation.

## DISCUSSION

The literature demonstrates a *deficit* in exercise capacity of cirrhotic patients when compared to the healthy population, both by means of the stress test and by the 6MWT. This is due to a circle of events involving liver disease that generated a decrease in the energy substrate, increasing the feeling of fatigue, leading to a reduction in the ADL and consequently, loss of more muscle mass.^(^ ^25^ ^-^ ^27^ ^)^

Recently it was questioned if the changes caused by HPS could worsen physical inactivity of liver patients, hindering even further the capacity for exercise, functional status, and respiratory muscle strength. In this way, the performance of cirrhotic patients with and with no HPS was compared, verifying that this hypothesis was relevant.^(^ ^20^ ^)^

Based on this idea, we considered that the cirrhotic patient with HPS reached the point of LTx in worse physical conditions; this could cause more immediate postoperative complications, as well as during the period of hospitalization.

In this study, the group of patients with HPS had a longer time of mechanical ventilation after LTx. However, it is important to discuss the reasons that led to such a result. Likewise patients with chronic obstructive pulmonary disease, those with HPS tend to have lower prior arterial oxygenation, especially those in our population, considering that, aiming at better diagnostic accuracy, the individuals chosen had P(A-a)O_2_ >20cmH_2_O. Since diagnosis of the syndrome is still an important challenge, many intensive care unit professionals have no knowledge of the patient’s prior situation, and in a conservative manner, they retard the weaning process, expecting arterial oxygenation normalization, which is not corrected immediately after the procedure. On the other hand, the greater functional and muscular compromise of patients with HPS, when compared to those with no HPS, can interfere in the tolerance to changing controlled ventilatory modes to spontaneous modes. Unfortunately, it was not possible in this study to identify which of these factors were preponderant in retarding the weaning process in the Group with Hepatopulmonary Syndrome.

The conditions were similar for the need of non-invasive ventilation. After extubation, HPS patients can present with lower arterial oxygenation values; in a conservative management, the professionals involved can opt to use this ventilatory support.

However, in this aspect, we were able to identify that of the 12 cases of noninvasive ventilation use among HPS patients, 10 were due to use of accessory muscles, and only two due to hypoxemia. On the other hand, among patients with no HPS, noninvasive ventilation was necessary in two cases due to use of accessory muscles. Extubation failed in only two cases, both in HPS patients. These data may suggest that perhaps the delay in the weaning process occurred more as a result of a worse functional and muscular condition − and consequent intolerance to the process of ventilatory mode progression − than of oxygenation alone. This worse functional and muscular condition of cirrhotic patients with HPS, when compared to the cirrhotic patients with no HPS, became evident when analyzing the performance of the two groups in the 6MWT, stress testing, and in manovacuometry. The consequences were longer intensive care unit stay and a longer hospital stay.

A recent literature review identified some interesting results related to prognosis of cirrhotic patients with HPS.^(^ ^19^ ^)^ A high mortality was noted in this population, when the same patients were candidates to LTx. Similar results were found when examining the period after this procedure, pointing to a survival of 10.6 months after surgery and that only 25% of those with PaO_2_<60mmHg survived 6 months. When compared to individuals with and with no HPS, those with the syndrome had a survival rate of 23% in 5 years, whereas those without the syndrome presented with a 63% survival rated during the same period. Nevertheless, it is important to point out that the primary objective of this study was to verify changes in the short-term after LTx, especially as to ventilatory support and hospital stay, with survival in second place, and as to this aspect, no data were found for comparison.

In this project, there was no difference in 30 day mortality after LTx. This result could be explained by the fact that this outcome is more related to intraoperative complications, such as hemorrhage and infections, and both groups were very similar as to ischemia time of the organ to be transplanted, presence of pulmonary hypertension, coagulation factors, and operative time. In this way, the assessment of mortality for a longer period becomes more important, during which a worse functional condition and the worse exercise capacity can play a more fundamental role.

There are limitations in this study, such as performance of physical tests during only one opportunity, and difficulty in collecting more data in a multidisciplinary environment like the intensive care unit; however, the results suggest that HPS may interfere in the pre-LTx condition of cirrhotic patients, and such fact may cause complications after this procedure, which demands special attention of the professionals involved in the unit.

Further studies should be conducted, considering it was difficult to discuss the results, and there is no study addressing the topic. Nonetheless, it seems relevant that a well-prepared intervention for cirrhotic patients, especially those with HPS, could enable them to be transplanted in better conditions, avoiding postoperative complications and even decreasing hospitalization costs.

## CONCLUSION

Cirrhotic patients with hepatopulmonary syndrome had longer mechanical ventilation time, need for noninvasive ventilation, longer intensive care unit and hospital stay.
